# PU.1 regulates *Ccr7* gene expression by binding to its promoter in naïve CD4^+^ T cells

**DOI:** 10.1002/2211-5463.12861

**Published:** 2020-04-30

**Authors:** Takuya Yashiro, Hiromi Takeuchi, Kazumi Kasakura, Chiharu Nishiyama

**Affiliations:** ^1^ Department of Biological Science and Technology Faculty of Industrial Science and Technology Tokyo University of Science Japan

**Keywords:** CCR7, Naïve CD4 T cells, PU.1

## Abstract

C‐C chemokine receptor type 7 (CCR7) is expressed on naïve T cells, B cells, and activated dendritic cells (DCs). We previously demonstrated that the transcription factor PU.1/*Spi1* positively regulates the expression of CCR7 in DCs. In the present study, we investigated the role of PU.1 in CCR7 expression in T cells. To confirm whether PU.1 is involved in the expression of CCR7, we conducted a ChIP assay in various T cells purified from splenocytes and thymocytes and found that PU.1 binds to the *Ccr7* promoter‐proximal region in spleen naïve CD4^+^ T cells, but not in thymocytes. Small interfering RNA‐mediated PU.1 knockdown resulted in decreased CCR7 expression in spleen naïve CD4^+^ T cells. Compared to naïve CD4^+^ T cells, *Spi1* and *Ccr7* mRNA levels decreased in Th1 and Th2 cells, in which PU.1 did not bind to the *Ccr7* promoter, suggesting that CCR7 expression decreases due to the dissociation of PU.1 from the *Ccr7* promoter during the development of effector T cells from naïve T cells. Collectively, we concluded that CCR7 expression level correlates with the binding level of PU.1 to the *Ccr7* promoter and PU.1 acts as a transcriptional activator of the *Ccr7* gene in naïve CD4^+^ T cells.

## Graphical abstract

According to the differentiation stages, T cells alter their localization by changing the expression of chemokine receptors. One of the chemokine receptors, CCR7, is highly expressed in naïve CD4^+^ T cells. In this article, we found that the transcription factor, PU.1, contributes to CCR7 expression by binding to its promoter in naïve CD4^+^ T cells.

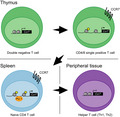

**Abbreviations**
CCR7: C‐C chemokine receptor type 7
DC: dendritic cell
TEC: thymic epithelial cell

Interactions between chemokines and chemokine receptors play critical roles in various aspects of immune responses by controlling cell migration. C‐C chemokine receptor type 7 (CCR7) is a G protein‐coupled receptor for CCL19 and CCL21 and is expressed on T cells, B cells, and activated dendritic cells (DCs) [1]. CCR7 expression drastically changes depending on the developmental stages and extracellular milieu.

T precursor cells initiate differentiation into T cells in the thymic cortex [2]. CD4^−^CD8^−^ (DN) thymocytes actively proliferate and express both CD4 and CD8. Each CD4^+^CD8^+^ (DP) thymocyte has different types of T‐cell receptor (TCR), but only cells with TCR recognizing major histocompatibility complex (MHC) molecules expressed on cortical thymic epithelial cell (cTEC) undergo survival and differentiation signals. Positively selected DP thymocytes differentiate into single‐positive cells expressing CD4 (CD4SP) or CD8 (CD8SP) and express CCR7. Subsequently, they migrate into the medulla by recognizing medullary TEC (mTEC)‐expressed CCL19 and CCL21. In the medulla, they interact with the self‐peptide–MHC complex presented by mTEC and DCs. T cells bearing TCR strongly reactive to self‐antigens are excluded by apoptosis in a process known as negative selection.

After exit from the thymus, naïve T cells migrate into secondary lymphoid organs in a CCR7‐dependent manner [3, 4]. CCR7 is also required for DC migration from the periphery to lymph nodes (LNs) upon capturing antigens [5]. Therefore, T cells can efficiently find a DC that presents its cognate antigen in the T‐cell zone of LNs, in which ligands of CCR7 are expressed by stromal cells and DCs [3]. Following antigen recognition, effector T cells express other homing molecules such as CCR4 (skin) and CCR9 (gut), which permit them to migrate to the source of their antigen in peripheral tissues. CCR7‐deficient mice and *plt/plt* mice, which are deficient for CCL19 and CCL21‐Ser, show significantly reduced numbers of thymocytes and naïve T cells in the thymus and LNs, respectively [6, 7, 8]. In addition, CCR7‐deficient mice tend to develop mild autoimmunity suggesting that this molecule plays important roles not only in adaptive immunity, but also in immune tolerance [9, 10, 11].

PU.1, encoded by *Spi1* gene, is a hematopoietic lineage‐specific transcriptional factor that plays essential roles in lymphoid and myeloid development by regulating numerous genes including the developmentally important cytokine receptors M‐CSFR, G‐CSFR, GM‐CSFRα, and IL‐7Rα [12, 13, 14, 15]. Recently, we have demonstrated that PU.1 is involved in CCR7 expression by binding to its promoter through −9/−6 TTCC in DCs [16]. Since PU.1 expression drastically changes at various T‐cell stages, we focused on the relationship between T‐cell differentiation and PU.1–CCR7 axis.

## Materials and methods

### Cell preparation

Spleen and thymus were obtained from 6‐ to 10‐week‐old BALB/c mice (Japan SLC, Hamamatsu, Japan). Naïve CD4^+^ T cells were isolated from splenocytes using a mouse Naïve CD4 T cell Isolation Kit and an autoMACS (all from Milteny Biotec, Tubingen, Germany). Naïve CD8^+^ T cells were isolated by using MojoSort Mouse CD8 Naïve T Cell Isolation Kit (BioLegend, San Diego, CA, USA). All animal experiments were performed according to the approved guidelines of the Institutional Review Board of Tokyo University of Science.

### Flow cytometric analysis

PE‐labeled anti‐CCR7 (4B12; BioLegend), FITC‐labeled anti‐CD4 (GK1.5; TONBO Bioscience, San Diego, CA, USA), and PE‐Cy5‐labeled anti‐CD8a (53–6.7; TONBO Biosciences) antibodies were used to stain cell‐surface molecules after blocking the Fc receptors with 2.4G2 (BD Pharmingen, Franklin Lakes, NJ, USA). In the experiment described in Fig. 2C, CD4^+^ T cells were fixed and permeabilized with Fixation and Intracellular Staining Permeabilization Wash Buffer (BioLegend). Fluorescence intensity was acquired by MACSQuant flow cytometry (Miltenyi Biotec, Tubingen, Germany) and analyzed by FlowJo (TOMY Digital Biology, Tokyo, Japan).

### Cell sorting

Thymocytes were sorted by Cell Sorter SH800 (Sony, Tokyo, Japan) after staining with FITC‐labeled anti‐CD4 and PE‐Cy5‐labeled anti‐CD8a antibodies.

### Small interfering RNA (siRNA) experiments

PU.1 (Stealth Select RNAi, *Sfpi1*‐MSS247676) and control (Stealth Negative Control) siRNAs were obtained from Thermo Fisher Scientific (Waltham, MA, USA). Naïve CD4^+^ T cells were activated by culturing in the presence of 1 µg·mL^−1^ plate‐bound CD3 and 10 µg·mL^−1^ soluble CD28. Activated CD4^+^ T cells were introduced to 200 pmol siRNA with a Neon Transfection System (Thermo Fisher Scientific) set at program 5.

### Quantitative RT–PCR

The total RNA was extracted using a ReliaPrep RNA Cell Miniprep System (Promega, Madison, WI, USA) according to the manufacturer’s instructions. cDNA was synthesized and amplified from 2 µg total RNA using a ReverTra Ace qPCR RT Kit (TOYOBO, Osaka, Japan). Quantitative real‐time PCR was performed using Thunderbird Probe qPCR Mix or Thunderbird SYBR qPCR Mix (TOYOBO) on a StepOne Real‐time PCR System (Applied Biosystems, Foster City, CA, USA). The TaqMan IDs for the genes analyzed are *mCcr7*, Mm01301785_m1; *mSpi1*, Mm00488142_m1; and *mGapdh*, 4352339E.

### Chromatin immunoprecipitation (ChIP) assay

ChIP assay was performed according to a previously described protocol [17]. Anti‐PU.1 antibody (D19; Santa Cruz Biotechnology, Santa Cruz, CA, USA) and goat IgG (Invitrogen, Carlsbad, CA, USA) were used. Quantitative PCR of chromosomal DNA was performed as described in subsection Quantitative RT‐PCR. The sequences of primer sets used were previously described [16].

### *In vitro* differentiation of Th1 and Th2

Naïve CD4^+^ T cells were cultured for 7 days with plate‐bound anti‐CD3ε and anti‐CD28 antibodies (both from TONBO Bioscience) in the presence of polarizing cytokines as follows: 10 ng·mL^−1^ IL‐12 (PeproTech) and 10 µg·mL^−1^ anti‐IL‐4 antibody (BioLegend) for Th1 cells and 20 ng·mL^−1^ IL‐4 (PeproTech) and 10 µg·mL^−1^ anti‐IL‐12 antibody (BioLegend) for Th2 cells.

### Western blotting

Western blotting was performed as previously described [18, 19].

### Statistical analysis

Data are expressed as mean + standard deviation (SD). Comparisons between multiple groups were analyzed with Tukey–Kramer test. The difference between two groups was analyzed by the unpaired Student’s *t*‐test. *P* values < 0.05 were considered statistically significant.

## Results

### PU.1 does not contribute to CCR7 expression during thymocyte development

**Fig. 1:**
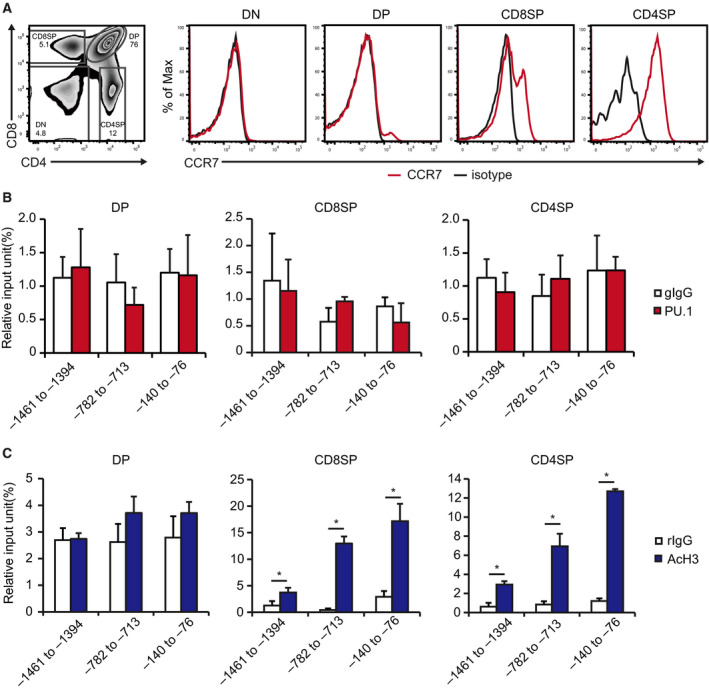
PU.1 is not involved in *Ccr7* gene expression during thymocyte development. (A) Thymocytes were stained with CD4‐FITC, CD8‐PECy5, and CCR7‐PE and analyzed by flow cytometry. Gates were placed around CD4^−^CD8^−^ (DN), CD4^+^CD8^+^ (DP), CD4^+^CD8^−^ (CD4SP), and CD4^−^CD8^+^ (CD8SP) cells. Representative histograms are shown. Similar results were obtained in three independent experiments. (B, C) Thymocytes were stained with CD4‐FITC and CD8‐PECy5, and sorted. ChIP assay was performed using either (B) goat IgG (gIgG) or anti‐PU.1 antibody (PU.1) or (C) rabbit IgG (rIgG) or anti‐acetyl histone H3 antibody (AcH3). The immunoprecipitated chromatin amount was determined by qPCR amplification of the indicated *Ccr7* promoter region. Data are expressed as a percentage of input for each ChIP assay. Results are presented as the mean + SD (DP and CD4SP; *n* = 5, CD8SP; *n* = 3). *P < 0.05, two‐tailed Student’s *t*‐test analysis.

During thymocyte development, CCR7 is required for the migration of positively selected thymocytes from the cortex to the medulla [20]. In our previous report, we demonstrated that PU.1 plays a central role in *Ccr7* gene expression in DCs [16]. We investigated whether PU.1 is involved in the *Ccr7* gene expression in thymocytes. Consistent with the results of previous studies [20, 21], CCR7 was expressed in CD4SP and CD8SP cells, but not in DN and DP cells (Fig. 1A). CD4SP cells expressed higher amount of CCR7 than CD8SP cells. We performed ChIP assay on sorted DP, CD4SP, and CD8SP cells. When we used the anti‐PU.1 antibody, there was no significant binding through the investigated region even at the most proximal region, where PU.1 apparently binds in DCs (Fig. 1B). These results indicate that PU.1 does not contribute to *Ccr7* gene expression via binding to its promoter during thymocyte development.

In addition to transcription factors, epigenetic regulation plays an essential role in gene expression through chromatin remodeling. Histone H3 tail acetylation is known to be the hallmark of transcriptional activation. To evaluate the histone H3 acetylation level at the *Ccr7* promoter, we carried out ChIP assay using the anti‐acetyl histone H3 antibody. As shown in Fig. 1C, histone H3 molecules in the promoter are highly acetylated in CD4SP and CD8SP cells, but not in DP cells. These results suggest that histone acetylation is induced in a PU.1‐independent manner both in CD4SP and CD8SP thymocytes.

### PU.1 transactivates Ccr7 gene in naïve CD4^+^ T cells

**Fig. 2:**
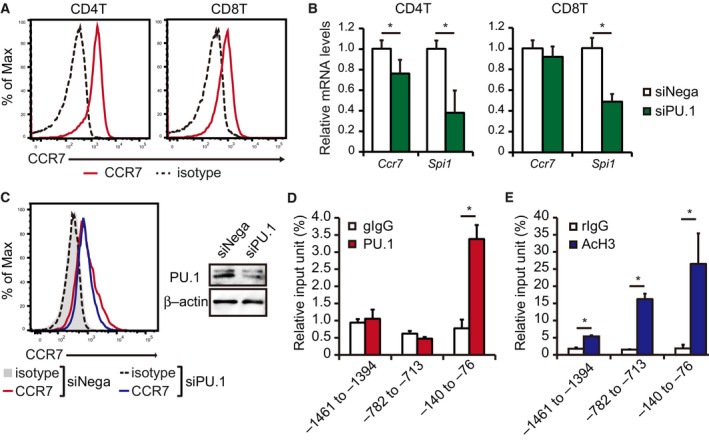
PU.1 is involved in *Ccr7* gene expression in naïve CD4^+^T cells from the spleen. (A) Representative histograms of CCR7 expression in CD4^+^ and CD8^+^ T cells from the spleen. Similar results were obtained in three independent experiments. (B, C) Naïve CD4^+^ or CD8^+^ T cells were cultured with plate‐bound anti‐CD3ε and anti‐CD28 antibodies. After 24 h of incubation, the cells were introduced with either negative control (siNega) or PU.1 (siPU.1) siRNA. (B) Relative mRNA levels were determined by quantitative RT–PCR and normalized to GAPDH mRNA levels. (C) Fixed and permeabilized CD4^+^ T cells were stained with CCR7‐PE and analyzed by flow cytometry. Representative histograms are shown. Similar results were obtained in two independent experiments. Protein levels of PU.1 and β‐actin were determined by western blotting. (D, E) ChIP assays were performed with naïve CD4^+^ T cells isolated from the spleen using either (D) gIgG or PU.1 or (E) rIgG or AcH3. The immunoprecipitated chromatin amount was determined by qPCR amplification of the indicated region of the *Ccr7* promoter. Data are expressed as a percentage of input for each ChIP assay. (B, D, E) Results are presented as the mean + SD (*n* = 3). *P < 0.05, two‐tailed Student’s *t*‐test analysis.

After differentiation in the thymus, naïve T cells locate to the T‐cell areas of lymph organs in a CCR7‐dependent manner. To confirm the CCR7 expression in splenic T cells, we performed flow cytometry and found that both CD4^+^ and CD8^+^ T cells highly expressed CCR7 (Fig. 2A). We introduced PU.1 siRNA into naïve CD4^+^ T cells and found that *Ccr7* mRNA level was slightly but significantly decreased by PU.1 knockdown (Fig. 2B left). As shown in Fig. 2C, similar results were obtained in the protein levels. On the other hand, PU.1 knockdown did not affect *Ccr7* mRNA level in naïve CD8^+^ T cells (Fig. 2B right). We next performed ChIP assay using an anti‐PU.1 antibody. When the primer set amplifying the most proximal region was used, the amount of chromosomal DNA immunoprecipitated with anti‐PU.1 antibody was much higher than that with the isotype control (Fig. 2D). However, there was no significant difference between the upstream‐region DNA immunoprecipitated with anti‐PU.1 antibody and isotype control, suggesting that PU.1 specifically binds around the transcription initiation site. These results indicate that PU.1, at least in part, is involved in *Ccr7* gene expression in naïve CD4^+^ T cells by binding to the promoter‐proximal region. To evaluate the histone H3 acetylation level at the *Ccr7* promoter, we carried out ChIP assay using an anti‐acetyl histone H3 antibody. As shown in Fig. 2E, histone H3 of the investigated regions was significantly acetylated, suggesting that histone acetylation contributes to the transcriptional activation in naïve CD4^+^ T cells.

### Expression of PU.1 and CCR7 was decreased after helper T‐cell differentiation

**Fig. 3:**
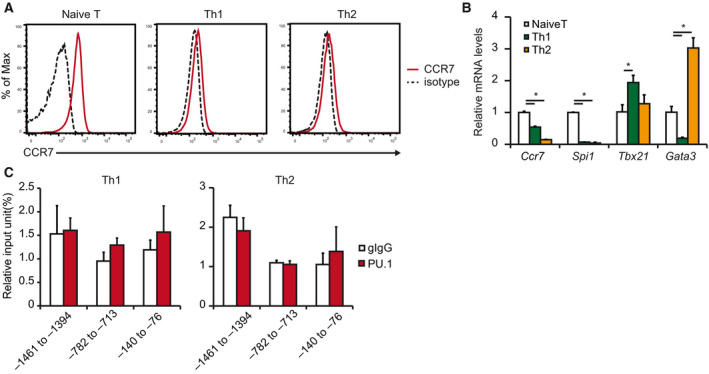
Relationship between CCR7 expression and PU.1–*Ccr7* promoter binding in helper T cells. (A–C) Naïve CD4^+^ T cells were cultured under Th1‐ or Th2‐polarizing conditions for 7 days. (A) Cells were stained with CCR7‐PE and analyzed by flow cytometry. Representative histograms are shown. Similar results were obtained in three independent experiments. (B) Relative mRNA levels were determined by quantitative RT–PCR after being normalized to GAPDH mRNA level. Data are expressed as a ratio to the mRNA expression levels in naïve T cells. (C) ChIP assay was performed using either gIgG or PU.1. The amount of immunoprecipitated chromatin was determined by qPCR amplification of the indicated *Ccr7* promoter region. Data are expressed as a percentage of input for each ChIP assay. (B, C) Results are presented as the mean + SD (*n* = 3). (B) *P < 0.05, Tukey–Kramer test. (C) *P < 0.05, two‐tailed Student’s *t*‐test analysis.

To investigate whether PU.1 is involved in *Ccr7* gene expression in helper T cells, we cultured naïve CD4^+^ T cells in polarizing conditions. The cell‐surface expression and the mRNA levels of *Ccr7* were significantly decreased in both Th1 and Th2 cells (Fig. 3A,B). In addition, *Spi1* mRNA level in these cells was markedly lower than naïve CD4^+^ T cells (Fig. 3B). Indeed, PU.1 did not bind to the *Ccr7* promoter in these cells (Fig. 3C). These results suggest that PU.1 expression level reduces after differentiation into helper T cells, thereby eliminating PU.1 contribution to *Ccr7* gene expression.

## Discussion

Proper regulation of CCR7 expression plays a critical role in T‐cell maturation, differentiation, and function. In the present study, we demonstrated that PU.1 positively regulates *Ccr7* gene expression in naïve CD4^+^ T cells. As in the case of DCs, this regulation was mediated by the binding of PU.1 to the *Ccr7* promoter‐proximal region. It has already been reported that FOXO1 is involved in *Ccr7* gene expression in naïve CD4^+^ T cells [22, 23]. However, CCR7 expression is not completely diminished by FOXO1 deficiency, suggesting the contribution of other transcription factors in the expression. We clearly demonstrated the involvement of PU.1 in *Ccr7* gene expression using siRNA against PU.1. However, we could not determine the level of the contribution because we were unable to utilize PU.1‐deficient mice due to their neonatal death [12, 13]. Since PU.1 is required for the optimal T‐cell development [24], we need to develop conditional PU.1 deficiency in mature T cells.

While PU.1 mRNA was observed in splenic T cells [25], previous reports using PU.1‐GFP reporter mice or PU.1 intracellular staining demonstrated that T cells isolated from spleen contain little or no PU.1 [26, 27, 28]. Considering that our data showed that PU.1 is involved in the CCR7 expression, PU.1 expression may be marginal but significant in naïve CD4^+^ T cells. In addition, it is possible that PU.1 regulates the expression of other genes in naïve CD4^+^ T cells because PU.1 is known to be involved in the transcription of numerous genes in DCs, macrophages, and B cells. In the future, the role of PU.1 in naïve CD4^+^ T cells will be revealed.

During thymocyte development, CCR7 is expressed at CD4SP and CD8SP cells, but PU.1 is not expressed at these cells [26, 28]. Consistent with this notion, our ChIP assay showed that PU.1 is not involved in *Ccr7* gene expression. Contrastingly, significant histone acetylation at the *Ccr7* promoter region was observed in both CD4SP and CD8SP thymocytes and sustained in spleen naïve CD4^+^ T cells. These results suggest that histone acetylation at the *Ccr7* promoter is controlled by a mechanism independent of PU.1‐binding to the promoter. Since histone acetylation is mediated by histone acetyltransferases (HATs), unknown transcription factor(s) recruiting HATs at the *Ccr7* promoter may exist in CD4SP and CD8SP cells.

After antigen recognition, CCR7 expression is diminished in activated T cells to egress from the LN and migrate into the infected area. PU.1 expression is reported to be decreased in Th1 and Th2 cells, but IL‐4 low Th2 subset still expresses PU.1, suggesting that PU.1 downregulation is required for adequate helper T‐cell differentiation [29]. Indeed, the expression of CCR7 and PU.1 was reduced in *in vitro* differentiated Th1 and Th2 cells. During differentiation from naïve T cells into effector T cells, PU.1 expression and PU.1‐binding to the *Ccr7* promoter might be suppressed followed by CCR7 downregulation.

Collectively, our results demonstrate that PU.1 is involved, although only moderately, in the *Ccr7* gene expression by binding to the promoter‐proximal region in naïve CD4^+^ T cells, but not in thymocytes and helper T cells.

## Conflict of interest

The authors declare no conflict of interest.

## Author contributions

TY designed research, performed experiments, analyzed data, and wrote the paper; HT performed experiments and analyzed data; KK provided experimental tools; and CN designed research.
